# Identification of m7G-Related miRNA Signatures Associated with Prognosis, Oxidative Stress, and Immune Landscape in Lung Adenocarcinoma

**DOI:** 10.3390/biomedicines11061569

**Published:** 2023-05-29

**Authors:** Sujing Jiang, Mingshu Xiao, Yueli Shi, Yongfang Wang, Zhiyong Xu, Kai Wang

**Affiliations:** 1 Department of Respiratory and Critical Care Medicine, The Fourth Affiliated Hospital, Zhejiang University School of Medicine, Yiwu 322000, China; 2 Second Affiliated Hospital, Zhejiang University School of Medicine, Hangzhou 310009, China

**Keywords:** N7-methylguanosine, lung adenocarcinoma, miRNA, immune, prognosis

## Abstract

The role of N7-methylguanosine(m7G)-related miRNAs in lung adenocarcinoma (LUAD) remains unclear. We used LUAD data from The Cancer Genome Atlas (TCGA) to establish a risk model based on the m7G-related miRNAs, and divided patients into high-risk or low-risk subgroups. A nomogram for predicting overall survival (OS) was then constructed based on the independent risk factors. In addition, we performed a functional enrichment analysis and defined the oxidative stress-related genes, immune landscape as well as a drug response profile in the high-risk and low-risk subgroups. This study incorporated 28 m7G-related miRNAs into the risk model. The data showed a significant difference in the OS between the high-risk and low-risk subgroups. The receiver operating characteristic curve (ROC) predicted that the area under the curve (AUC) of one-year, three-year and five-year OS was 0.781, 0.804 and 0.853, respectively. The C-index of the prognostic nomogram for predicting OS was 0.739. We then analyzed the oxidative stress-related genes and immune landscape in the high-risk and low-risk subgroups. The data demonstrated significant differences in the expression of albumin (ALB), estimated score, immune score, stromal score, immune cell infiltration and functions between the high-risk and low-risk subgroups. In addition, the drug response analysis showed that low-risk subgroups may be more sensitive to tyrosine kinase inhibitor (TKI) and histone deacetylase (HDAC) inhibitors. We successfully developed a novel risk model based on m7G-related miRNAs in this study. The model can predict clinical prognosis and guide therapeutic regimens in patients with LUAD. Our data also provided new insights into the molecular mechanisms of m7G in LUAD.

## 1. Introduction

Lung cancer is the most common malignancy and a leading cause of cancer-specific deaths worldwide, which is majorly attributed to an advanced diagnosis at advanced or inoperable stages [1]. Previous data have shown that identifying operable alterations in oncogenic drivers and immune checkpoints leads to significant improvement in the outcome of patients with advanced non-small-cell lung cancer (NSCLC) treated with biomarker-driven therapy and immunotherapy [2]. Lung adenocarcinoma (LUAD), the most common subtype of NSCLC, has a poor survival rate and high heterogeneity, despite improved standard treatment strategies based on clinical stage [3]. Therefore, there is a need to develop a method to stratify risk and guide treatment of LUAD accurately.

Epigenetic alterations refer to heritable genetic alterations rather than mediation by changes in a DNA sequence, which have been shown to contribute to phenotypic composition and carcinogenesis over the past decade [4,5]. Although somatic genetic aberrations, such as copy number variations and mutations, play a vital role in tumorigenesis, data have shown that epigenetic alterations occur more frequently, and significantly, compared to somatic genetic aberrations [6].

RNA modification is one type of epigenetic alteration, in which RNA methylation accounts for about 50%, and plays an essential role in regulating gene functions [7]. Modified forms, such as N6-methyladenosine (m6A), N1-methyladenosine (m1A), N7-methylguanidine (m7G), 5-methylcytidine (m5C) and 2′-O-methylation, are dominant in RNA methylation and are widely present in various types of RNAs [8,9]. m7G, the most conserved modified nucleoside, is co-transcriptionally added to the 5′ cap structure in eukaryotes and a few archaea [10,11]. This significant cap-like modification joins almost every phase of mRNA regulation, which includes transcription stabilization [12], pre-mRNA splicing [13], polyadenylation [14], nuclear export [15], and translation [16].

Meanwhile, the m7G RNA modification is also involved in the functional regulation of rRNA [17,18] and tRNA [19,20]. Previous data have shown that the m7G modification of 18s RNA-specific G1639 site is triggered by BUD23/TRMT112 methyltransferase complex [21], which actively induces 18S rRNA precursor biogenesis [22], while the modification of tRNAs is mediated by the methyltransferase-like 1 (METTL1) and WD repeat domain 4(WDR4) complex [23], which was identified as an m7G writer [24]. Furthermore, RAM and RNA guanine-7 methyltransferase (RNMT) form an m7G-modified enzyme complex, which protects RNA from cleavage by exonuclease and affects RNA processing, export, and translation [25]. These m7G-regulated genes promote m7G modification of targeted RNA molecules and play a double-edged role in various cancers by regulating the expression of tumor-related genes to facilitate or inhibit oncogenesis [26].

Non-coding RNA plays an important role in tumor development and in the regulation of immune cell function [27]. Among them, microRNA (miRNA) has been widely recruited in regulating the expression of oncogenes and tumor-suppressor genes at multiple levels, including in transcription, post-transcription, epigenetics and post-translation [28]. It has been reported that the analysis of miRNAs in plasma, serum and sputum is a noninvasive biomarker for the early detection of NSCLC [29]. Notably, the obtained miRNA samples do not show any tendency towards degradation when properly stored and handled, which is an advantage in clinical applications [30]. Recent data have demonstrated that METTL1 could methylate let-7e miRNA precursor in lung cancer cells, consequently inhibiting the progression and invasion of lung cancer [31]. The methylation regulates the expression of key oncogenes such as *RAS, Myc* and the high-mobility group AT-hook 2 (*HMGA2*). However, the mechanisms and responses of miRNAs and m7G in lung cancer, especially LUAD, have not been fully elucidated.

Oxidative stress is usually caused by an imbalance between the production and elimination of reactive oxygen species (ROS) [32]. The high levels of ROS can affect alteration in cellular metabolic signaling pathways, especially in tumor cells [33]. It has been reported that the levels of oxidative stress increase in lung cancer [34,35]. Nevertheless, the connection with m7G-related miRNAs and oxidative stress remains unclear.

Here, we first focused on how m7G-related miRNAs were expressed in LUAD. We discovered several significant m7G-related miRNAs that may serve as LUAD prognostic factors. Next, we conducted an additional study on the m7G-related miRNAs found in LUAD to determine their relationships to oxidative stress, the immune system, and medication responsiveness. Overall, our thorough study revealed the regulation mechanism of m7G-related miRNAs, opening up a fresh viewpoint for a better comprehension of the function of m7G in LUAD.

## 2. Materials and Methods

### 2.1. Data Collection

We profiled miRNA expressions in 521 LUAD patients and 46 normal patients, mRNA expression in 539 LUAD patients and 59 normal patients, and selected the clinical information of 455 LUAD patients from the Cancer Genome Atlas (TCGA) (https://portal.gdc.cancer.gov/, accessed on 26 July 2022). Patients with other malignancies and incomplete survival data were excluded.

### 2.2. Screening of m7G-Related Genes and Prediction of m7G-Related miRNAs

Based on the literature search, we identified m7G-related genes which included *BUD23, TRMT112, METTL1, WDR4, RAM* and *RNMT*. We then used the online analysis tools TargetScan (https://www.targetscan.org/vert_72/, accessed on 2 August 2022) to predict the target m7G-related miRNAs.

### 2.3. Screening of Oxidative Stress-Related Genes

Oxidative stress-related genes were obtained from the genecards website (https://www.genecards.org/, accessed on 2 August 2022) with a relevance score more than 20.

### 2.4. Differential Expression Analysis

The miRNA expression was analyzed using R software packages edgeR [36] and limma [37], which were used to identify m7G-related differentially expressed miRNAs (DEMs). The cutoff criteria were *p*.adjust < 0.05 and |log2FC|> 1.0. We then used the online software, miEAA 2.0 (https://ccb-compute2.cs.uni-saarland.de/mieaa2/, accessed on 3 August 2022) [38], to enrich disease pathways associated with the m7G-related DEMs.

### 2.5. Construction and Validation of a Prognostic m7G-Related DEMs

To screen for m7G-related DEMs with prognostic value, a univariate Cox analysis was performed on overall survival (OS). To avoid overfitting, a least absolute shrinkage and selection operator (LASSO) regression was employed to construct the prognostic model using the R software package “glmnet” [39]. The penalty parameter λ of the model was determined by ten-fold cross-validation, according to the minimum criterion. In addition, based on the optimal lambda value, the risk-prediction model and risk formula were established by selecting the best possible m7G-related miRNAs. The risk scores of each patient were calculated based on the corresponding regression coefficients and normalized expression of each screened m7G-related miRNA. The patients were then divided into high-risk or low-risk subgroups according to the median risk score. On the other hand, a time-dependent receiver operating characteristic curve (ROC) was performed using the “timeROC” R package to assess the predictive power of the m7G-related DEMs signature.

### 2.6. Establishment of a Predictive Nomogram and Calibration

To investigate whether age, sex, stage, risk score or risk level of the m7G-related miRNAs can independently predict prognosis in patients with LUAD, univariate and multivariate Cox regression analyses were performed for all 5 variables. Those variables with a *p* < 0.05 in both the regression analyses were considered as independent risk factors. A nomogram for the 1-, 2-, and 3-year OS was constructed based on the independent risk factors. The C-index and calibration curves based on the Hosmer–Lemeshow test were used to evaluate the accuracy of the nomogram.

### 2.7. Functional Enrichment Analysis

Edger and limma R packages were used to analyze the differential gene expression (DGE) between the high-risk and low-risk subgroups. We then performed Gene Ontology (GO), Kyoto Encyclopedia of Genes and Genomes (KEGG) pathway and gene set enrichment analysis (GSEA). A p value corrected false by discovery rate (FDR) less than 0.05 was considered significant.

### 2.8. Tumor Microenvironment and Immune Landscape Analysis

The estimated score, immune score, and stromal score were calculated using the ESTIMATE algorithm via “estimate” R package [40]. The differences in the score between the high-risk and low-risk subgroups were compared to define the status of the tumor microenvironment (TME). The degree of infiltration of 28 immune cells in the TME and immune function were quantitatively analyzed by the ssGSEA method [41]. In addition, we employed the “ggpubr” R package to assess the immune checkpoints between the low-risk and high-risk subgroups.

### 2.9. Protein Expression and Prognostic Power of Oxidative Stress-Related Genes in LUAD

The Human Protein Atlas (https://www.proteinatlas.org/, accessed on 20 August 2022) is a widely used database on the basis of immunohistochemistry (IHC), which can prompt protein expression in normal tissues and in pathological tissues [42]. In this study, the protein expression of oxidative stress-related genes in clinical specimens from LUAD patients were obtained from this database. The relationship between the expression of oxidative stress-related genes and the OS of LUAD was acquired from the Kaplan–Meier Plotter database (http://kmplot.com/analysis/, accessed on 20 August 2022).

### 2.10. RNA Isolation

Four pairs of human LUAD samples along with the adjacent normal tissue samples were obtained from the Second Affiliated Hospital of Zhejiang University (approval No. 20210656; approval on 28 June 2021). The RNA-Quick purification kit (RN001, ESscience, Shanghai, China) was utilized to isolate RNA from the tissues.

### 2.11. Quantitative Real-Time PCR

Hiscript III All-in-one RT SuperMix (R333-01, Vazyme, Nanjing, China) was employed to convert the RNA into cDNA. ChamQ Universial SYBR qPCR Master Mix (Q711-02, Vazyme, China) was used for the quantification of the real-time PCR analyses and normalized according to GAPDH levels. The primers used were as follows: GAPDH forward, ACAACTTTGGTATCGTGGAAGG; GAPDH reverse, GCCATCACGCCACAGTTTC; ALB forward, TGCAACTCTTCGTGAAACCTATG; ALB reverse, ACATCAACCTCTGGTCTCACC.

### 2.12. Subgroup Analyses of Drug Response

To investigate the sensitivity of drug therapy, the R package “oncoPredict” was employed to predict the concentration which suppresses growth by 50% (IC_50_) in both the high-risk and low-risk subgroups [43]. IC_50_ represents the ability of a substance to inhibit a specific biological or biochemical function. Differences between the subgroups were tested using the t-test. RNAactDrug database (http://bio-bigdata.hrbmu.edu.cn/RNAactDrug, accessed on 11 May 2023), which is used to predict the m7G-related miRNAs associated with drug sensitivity.

### 2.13. Statistical Analysis

The gene expression in LUAD and normal tissues was compared using the Student’s t-test. The OS between the high-risk and low-risk subgroups was compared by Kaplan–Meier analysis, with the log-rank test. Univariate and multivariate Cox regression analyses were used to identify independent predictors of OS. All statistical analyses were performed using R software (Version 4.1.2). If not specified above, a *p* < 0.05 was considered statistically significant and all *p* values were two-tailed.

## 3. Results

### 3.1. Patient Characteristics

**Figure 1:**
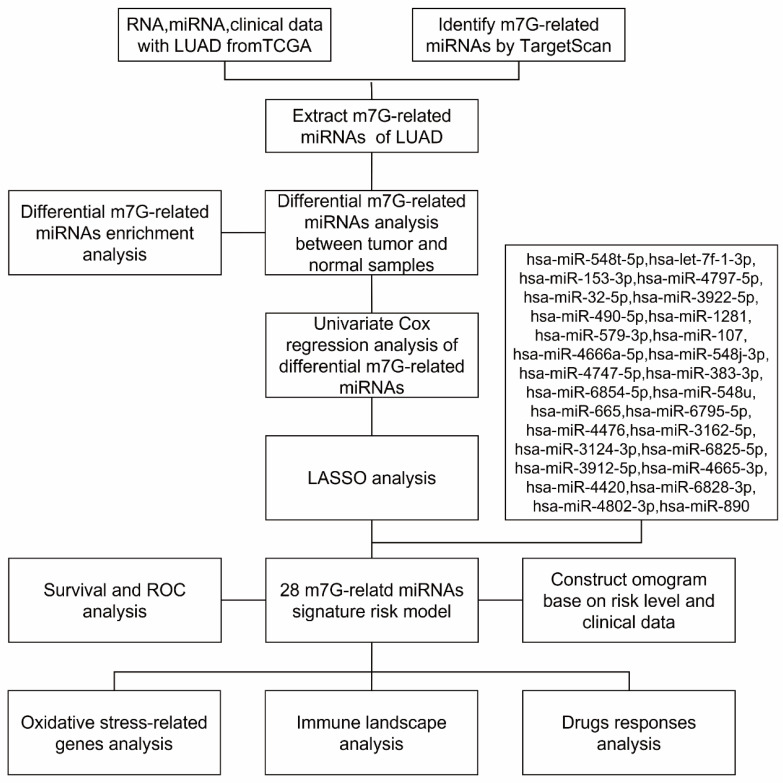
The research flowchart.

The miRNA expression of 521 LUAD patients and 46 normal patients, mRNA expression of 539 LUAD patients and 59 normal patients, and clinical information of 455 LUAD patients were selected from the TCGA and analyzed. Overall, our data showed that 249 (54.7%) LUAD patients were diagnosed at stage I, 108 (23.7%) at stage II, 74 (16.3%) at stage III and 24 (5.3%) at stage IV. Patients with T2 disease were the most numerous (52.1%), followed by patients with T1 disease (35.2%), T3 disease (8.4%), T4 disease (3.7%) and Tx disease (0.6%). A total of 151 (33.2%) patients with LUAD had lymph node metastasis, while 294 (64.6%) patients had no lymph node metastasis. In addition, 308 (67.7%) patients did not have distant metastasis. Supplementary Table S1 summarizes the detailed clinical characteristics of these patients. The research flowchart is as shown in Figure 1.

### 3.2. Identification of m7G-Related miRNAs in LUAD

Our analysis demonstrated that there were significant differences in the expression of m7G-related genes *BUD23* (*p* < 0.001), *TRMT112* (*p* < 0.001), *METTL1* (*p* < 0.001), *WDR4* (*p* < 0.001), *RAM* (*p* < 0.001) and *RNMT* (*p* < 0.001) between the LUAD and normal samples, and all of them were highly expressed in tumor tissues (Supplementary Figure S1). We then explored the regulators that can affect the expression of these genes. A total of 3106 miRNA/mRNA regulatory pairs predicted by the TargetScan were analyzed.

### 3.3. Differentially Expressed and Functional Analysis of the m7G-Related miRNAs

**Figure 2:**
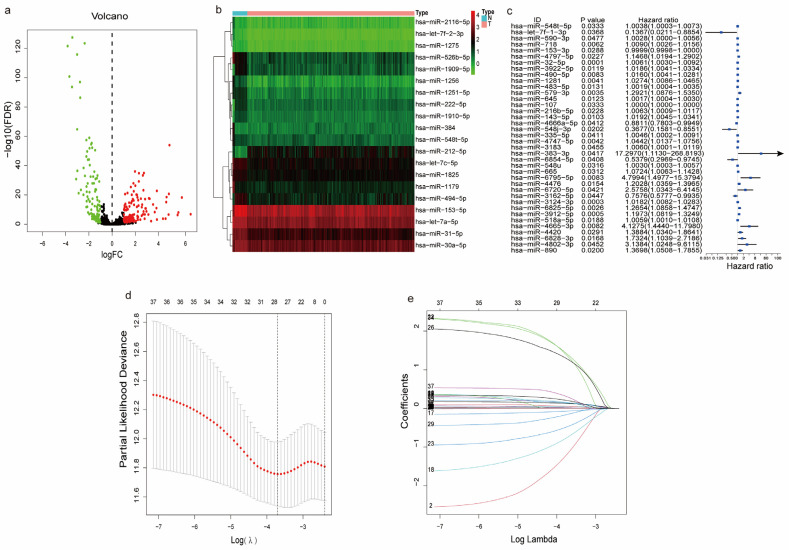
Functional analysis of differentially expressed m7G-related miRNAs: (**a**) volcano showing expression of m7G-related DMEs in LUAD and normal samples; the green dots represent the downregulated genes, while red dots represent the upregulated genes; (**b**) heatmap of the top 20 differentially expressed m7G-related miRNAs; (**c**) forest plots showing the results of the univariate Cox regression analysis for m7G-related DEMs and OS; (**d**) penalty plot of the LASSO model for the 28 prognostic m7G-related DEMs with error bars denoting standard errors; and (**e**) LASSO coefficient profile plots showing that variations in the size of coefficients for parameters shrank as the value of the k penalty increased.

A total of 269 out of the 3106 m7G-related miRNAs were significantly differentially expressed between the LUAD and normal samples. Out of these, 149 m7G-related miRNAs were upregulated, while 120 miRNAs were downregulated (Figure 2a). As shown in Figure 2b, our heatmap showed the top 20 differentially expressed m7G-related miRNAs. We then analyzed the m7G-related DME enrichment pathways using the online miEAA database and showed that the m7G-related DMEs were enriched in many tumors, including LUAD (Supplementary Figure S2).

### 3.4. Predictive Ability of the Risk Score

A univariate Cox regression analysis was used to evaluate the prognostic relationship between the m7G-related DMEs and OS in LUAD. A total of 38 prognostic genes were identified (Figure 2c). To prevent overfitting of the prognostic model, we performed LASSO Cox regression analysis (Figure 2d,e). The analysis showed that 28 genes were incorporated into the risk score formula with minimized lambda as follows:

Risk score = hsa-miR-548t-5p × 0.001095 − hsa-let-7f-1-3p × 1.14684 − hsa-miR-153-3p × 5.91 × 10^−5^ + hsa-miR-4797-5p × 0.048328 + hsa-miR-32-5p × 0.004674 + hsa-miR-3922-5p × 0.007469 + hsa-miR-490-5p × 0.012056 + hsa-miR-1281 × 0.011879 + hsa-miR-579-3p × 0.135198 + hsa-miR-107 × 8.31 × 10^−7^ − hsa-miR-4666a-5p × 0.06724 − hsa-miR-548j-3p × 0.73184 + hsa-miR-4747-5p × 0.011799 + hsa-miR-383-3p × 1.334684 − hsa-miR-6854-5p × 0.46268 + hsa-miR-548u × 0.000565 + hsa-miR-665 × 0.021734 + hsa-miR-6795-5p × 1.26435 + hsa-miR-4476 × 0.053603 − hsa-miR-3162-5p × 0.14938 + hsa-miR-3124-3p × 0.008619 + hsa-miR-6825-5p × 0.14571 + hsa-miR-3912-5p × 0.127381 + hsa-miR-4665-3p × 1.381697 + hsa-miR-4420 × 0.143256 + hsa-miR-6828-3p × 0.149028 + hsa-miR-4802-3p × 0.290423 + hsa-miR-890 × 0.201132.

**Figure 3:**
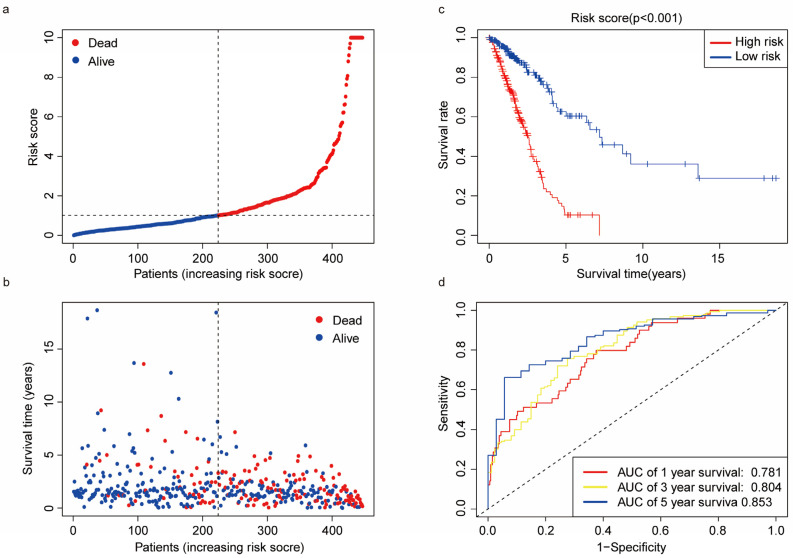
Prognostic analysis of the risk score model: (**a**) distribution of risk scores among LUAD patients; (**b**) the survival time and survival status among high-risk and low-risk subgroups; (**c**) Kaplan–Meier survival curve for the OS of patients in the high-risk and low-risk groups; and (**d**) time-related ROC analysis demonstrating the prognostic performance of the risk score.

According to the median cutoff value, patients were categorized into high-risk and low-risk subgroups (Figure 3a,b). The Kaplan–Meier survival analysis demonstrated that the OS was higher in the low-risk group compared to that of the high-risk group (Figure 3c). The ROC predicted that the area under curve (AUC) of one-year, three-year, and five-year OS was 0.781 (95% CI 0.7143–0.8483), 0.804 (95% CI 0.7440–0.8639), and 0.853 (95% CI 0.7846–0.9204), respectively (Figure 3d).

### 3.5. Nomogram Construction and Calibration

**Figure 4:**
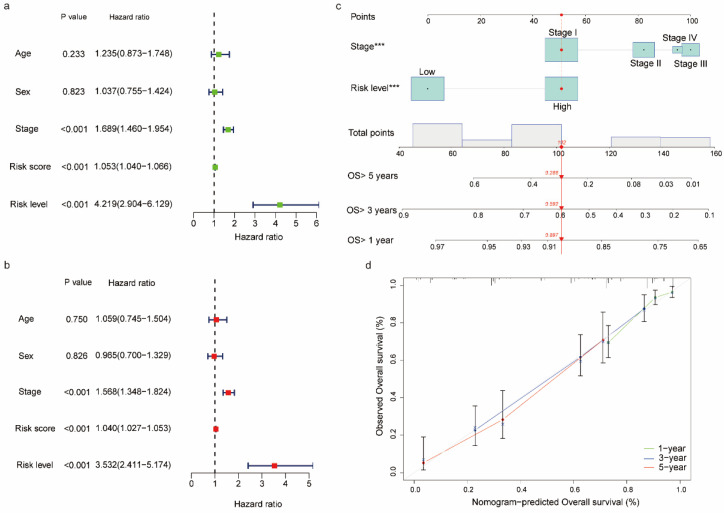
Nomogram for predicting the probability of one-, three- and five-year OS in LUAD patients: (**a**) forest plot showing the univariate regression analyses for the clinical characteristics, risk scores, and risk level for the high-risk and low-risk subgroups; (**b**) forest plot showing the multivariate regression analyses for clinical characteristics, risk scores, and risk level for the high-risk and low-risk subgroups; (**c**) a nomogram integrating the tumor stage and risk level; and (**d**) calibration plot of the nomogram depicting the agreement between predicted and observed outcomes. *** *p* < 0.001.

To determine the independent prognostic indicators of OS in LUAD, univariate and multivariate cox regression analyses were performed based on the clinical characteristics, risk scores and risk levels. As shown in Figure 4a, tumor stage (HR 1.689; 95% CI 1.460–1.954; *p* < 0.001), risk score (HR 1.0530; 95% CI 1.040–1.066; *p* < 0.001), and risk level (HR 4.219; 95% CI 2.904–6.129; *p* < 0.001) were independent prognostic parameters in LUAD and showed that the risk-discrimination degree of the risk level was better than risk score. In addition, the data demonstrated that age (HR 1.235; 95% CI 0.873–1.748; *p* = 0.233) and sex (HR 1.037; 95% CI 0.755–1.424; *p* = 0.823) were not prognostic factors for OS in LUAD. The multivariate cox regression analysis showed similar results (Figure 4b). Subsequently, to provide clinicians with a quantitative method to predict the prognosis of patients with LUAD, we constructed a nomogram which was based on the risk level and tumor stage to predict one-, three-, and five-year OS in LUAD patients. As shown in Figure 4c, each parameter was scored according to its prognostic value, and the total score could be used to estimate one-, three- and five-year survival probability. Furthermore, a calibration curve was developed to evaluate the performance of the nomogram (Figure 4d). The data showed that the predicted OS was like the observed outcome, which indicated that the prediction model had a high predictive consistency. Notably, the C-index of the prognostic nomogram for predicting OS was 0.739 (95% CI 0.692–0.786; *p* < 0.001), suggesting that the nomogram prediction based on risk level and tumor stage exhibited an excellent performance in predicting OS in LUAD patients.

### 3.6. Functional Enrichment Analysis

**Figure 5:**
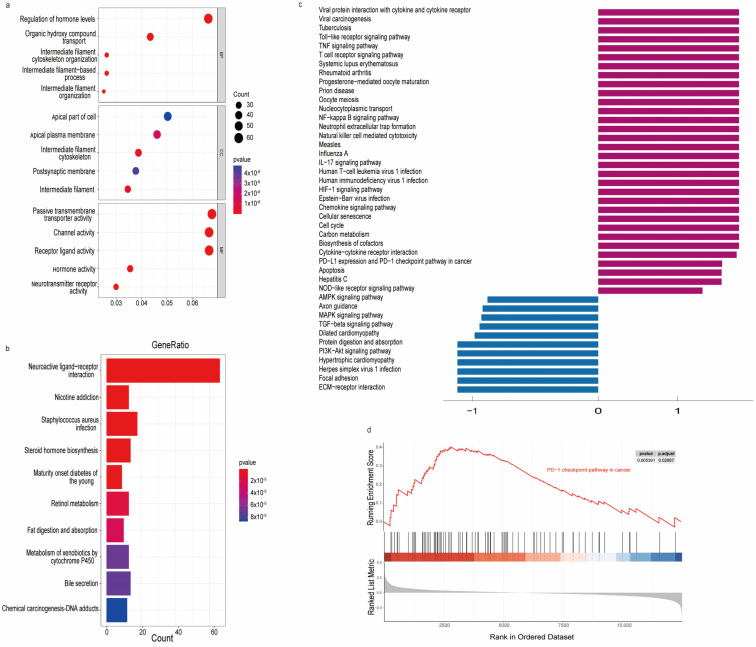
Functional enrichment analysis for the high-risk and low-risk subgroups. (**a**) GO analysis for the high-risk and low-risk subgroups; (**b**) KEGG analysis for the high-risk and low-risk subgroups; (**c**) GSEA of the high-risk and low-risk subgroups; and (**d**) differential PD-L1 expression and PD-1 checkpoint pathway enrichment between high-risk subgroups and low-risk subgroups.

To better understand the potential biological functions of the m7G-related DEMs, we identified the top 10 GO categories (Figure 5a) and KEGG analysis (Figure 5b) with significant enrichment between the high-risk and low-risk subgroups. GO functional annotation involves the biological process (BP), cellular component (CC), and molecular function (MF), which are most significantly involved in intermediate filament organization, and intermediate filament organization and hormonal activities, respectively. In the KEGG enrichment analysis, the altered m7G-related DEMs were mostly associated with neuroactive ligand−receptor interaction. To identify oncogenic signals, GSEA was performed according to the upregulated and downregulated genes in the differentially expressed genes in the high-risk and low-risk subgroups (Figure 5c). As shown in Figure 5d, the PD-1 checkpoint pathway in cancer was significantly different between the high-risk and low-risk subgroups.

### 3.7. The Immune Landscape between the High and Low-Risk Groups

**Figure 6:**
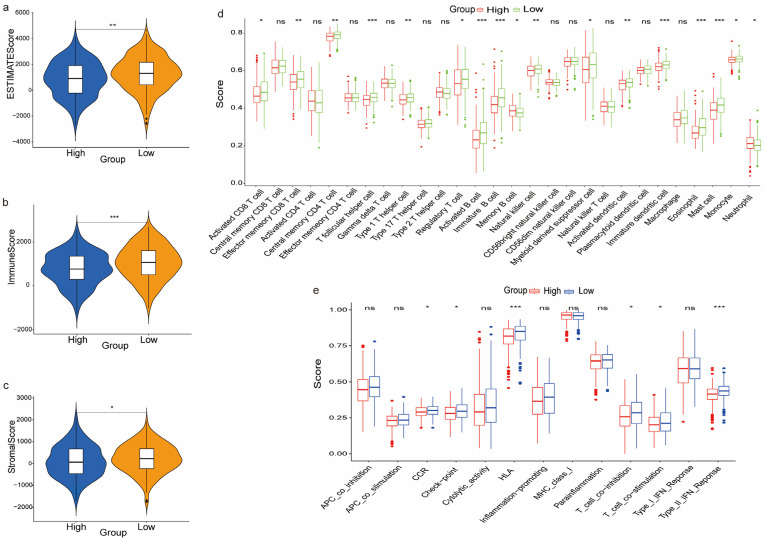
The immune landscape for the high and low-risk groups: (**a**) the estimated score between the high-risk and low-risk groups; (**b**) the immune score for the high-risk and low-risk groups; (**c**) the stromal scores for the high-risk and low-risk groups; (**d**) comparison of infiltration scores of 22 immune cell types between high-risk and low-risk groups; and (**e**) comparison of immune function scores between high-risk and low-risk groups. * *p* < 0.05, ** *p* < 0.01 and *** *p* < 0.001.

**Figure 7:**
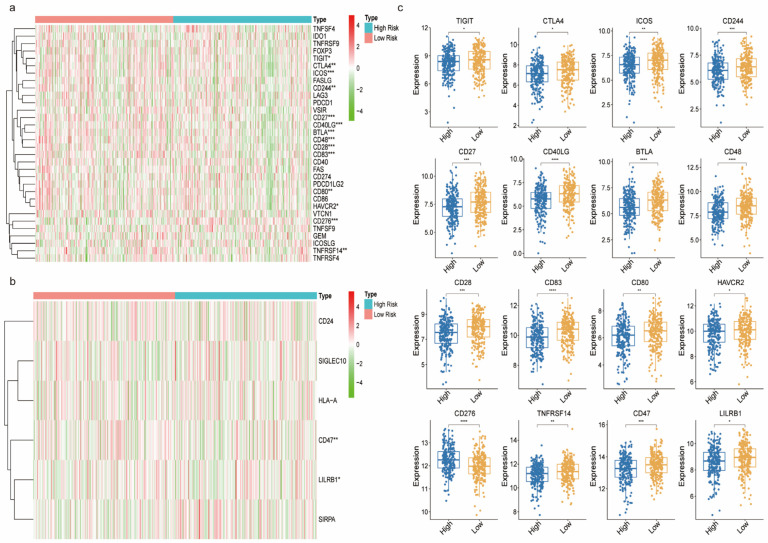
Comparison of immune checkpoint between high-risk and low-risk groups: (**a**) the T-cell-related immune checkpoints for the high-risk and low-risk subgroups; (**b**) macrophage-related immune checkpoints; and (**c**) the boxplot showing all immune checkpoints. * *p* < 0.05, ** *p* < 0.01, *** *p* < 0.001 and **** *p* < 0.0001.

We then evaluated the estimated, immune, and stromal scores between the high-risk and low-risk subgroups (Figure 6a–c). The analysis demonstrated that all three scores were lower in the high-risk patients compared to the low-risk group. We then performed an ssGSEA analysis to examine the correlation between different risk levels, immune cell infiltration and immune function. The boxplots of the tumor immune cell distribution in the two subgroups are shown in Figure 6d. The results showed that the low-risk subgroup had significantly higher proportions of activated CD8^+^ T cells, effect or memory CD8^+^ T cells, central memory CD4^+^ T cells, T follicular helper cells, type 1 T helper cells, regulatory T cells, activated B cells, immature B cells, natural killer cells, myeloid-derived suppressor cells, activated dendritic cells, immature dendritic cells, eosinophils, mast cells, and monocytes, neutrophils, while there was a higher infiltration of memory B cells in the high-risk group. Analysis of the correlation between immune cell subsets and related functions was performed by ssGSEA and showed a significant difference in the CCR, checkpoint, HLA, T cell co-inhibition, T cell co-simulation and type II IFN response between the high-risk and low-risk subgroups (Figure 6e). Given the importance of immunotherapy based on immune checkpoint inhibitor (ICI), we analyzed the expression of immune checkpoints between the two groups. The analysis showed that there was a significantly different expression of T cell-related immune checkpoints: *CTLA4, BTLA, TIGIT, CD27, TNFRSF14, CD28, ICOS, HAVCR, CD244, CD80, CD48, CD83, CD276, CD40LG* (Figure 7a); and macrophage-related immune checkpoints: *CD47* and *LILRB1*, between the two subgroups of patients (Figure 7b). The boxplot shows the different immune checkpoints (Figure 7c).

### 3.8. The Oxidative Stress-Related Genes between the High and Low-Risk Groups

**Figure 8:**
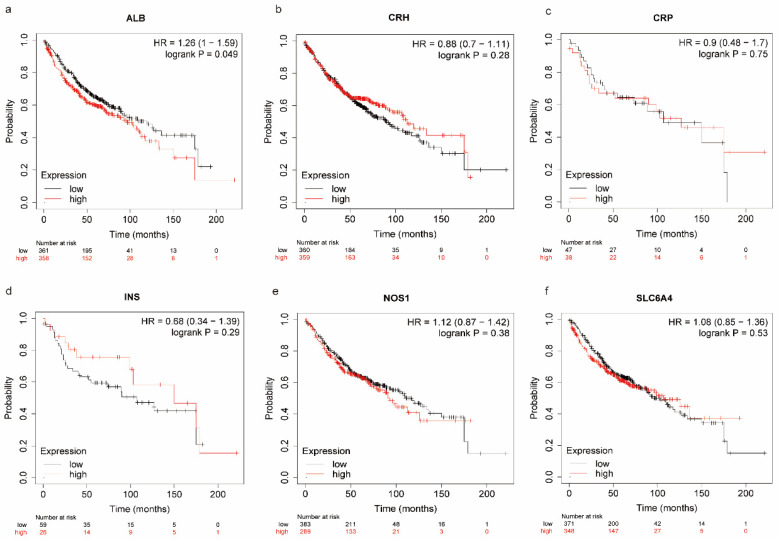
The survival analysis of LUAD patients with different expressions of ALB (**a**); CRP (**b**); CRH (**c**); INS (**d**); NOS1 (**e**); and SLC6A (**f**), respectively.

A total of 83 oxidative stress-related genes were screened out from gene cards with a relevance score of more than 20 (Supplementary Table S3). Among them, *NOS1, ALB, CRP*, *SLC6A, CRH* and *INS* were differently expressed between the high and low-risk groups. Moreover, the highly expressed *ALB* showed poor prognosis, while the expression of *NOS1, CRH, CRP, INS*, and *SLC6A4* showed no significant correlation with the prognosis of LUAD (Figure 8a–f).

### 3.9. The Protein Expression Levels of Oxidative Stress-Related Genes in LUAD Tissues

**Figure 9:**
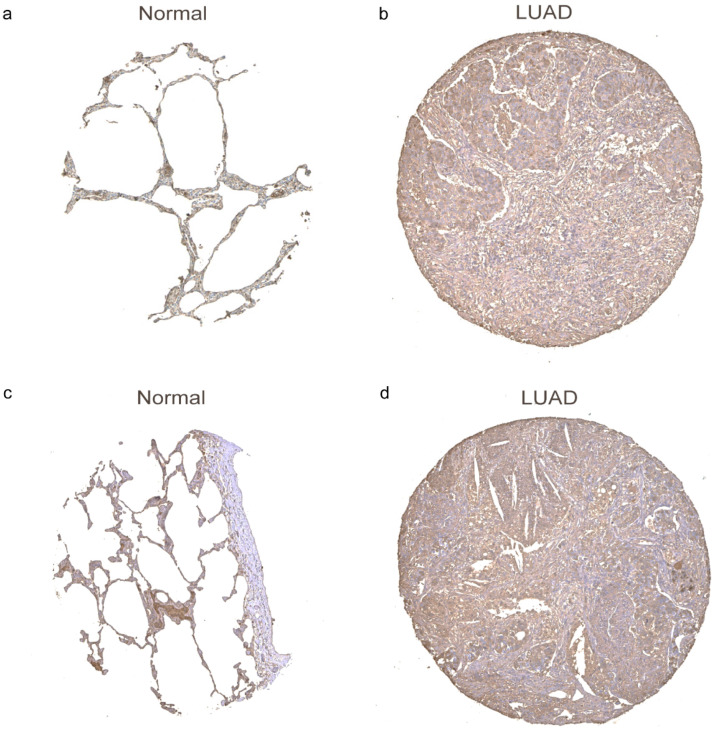
The protein expression of ALB between the normal tissue (**a**,**c**); and LUAD (**b**,**d**).

Considering that *ALB*, as oxidative stress-related genes, was associated with the prognosis of LUAD, we further investigated the expression of ALB in LUAD and normal tissues via the HPA database. Moreover, ALB were highly expressed in normal lung tissues (Figure 9a,c) than that in LUAD tissues (Figure 9b,d).

### 3.10. The Oxidative Stress-Related Genes Validation in LUAD Patients

**Figure 10:**
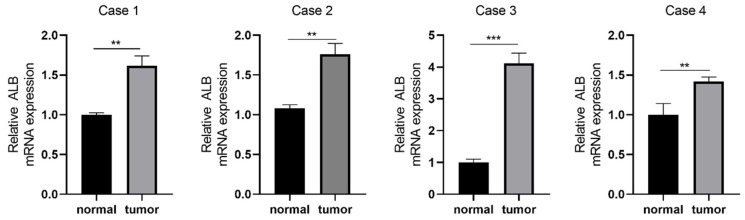
The mRNA expression of ALB between the normal tissue and LUAD. ** *p* < 0.01 and *** *p* < 0.001.

We then exerted qPCR to verify the expression patterns of *ALB* in LUAD and adjacent tissues. The results demonstrated that the *ALB* were significantly overexpressed in LUAD (Figure 10).

### 3.11. Drug Responses

**Figure 11:**
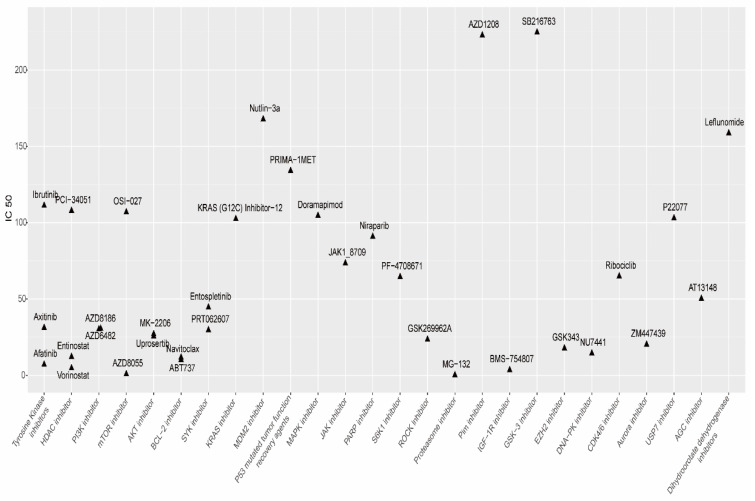
The 25 categories of sensitive drugs in the low-risk subgroup. The vertical axis represents IC50, the horizontal axis represents the drug classification. The position of each point on the graph represents the corresponding IC50 and drug classification, respectively.

We further evaluated drug response of patients with LUAD between the high-risk and low-risk subgroups. Our data demonstrated that there were 35 sensitive drugs in the low-risk subgroup. The drugs were classified into 25 categories, and tyrosine kinase inhibitor (TKI), histone deacetylase (HDAC) inhibitors, PI3K/AKT/mTOR pathway inhibitors and Bcl-2 apoptosis inhibitors showed lower IC_50_, suggesting that related signaling pathways had the potential to regulate the expression of m7G-related miRNAs (Figure 11).

## 4. Discussion

LUAD is a solid tumor that mainly originates from the distal airway with high tumor heterogeneity [44,45]. In the current era of precision medicine, there is an urgent need to establish a more accurate method to evaluate prognosis and guide the treatment of LUAD patients. Previous data have shown that modification of m7G may be involved in translational regulation of cancer development [46]. However, the cause of dysfunctional m7G regulator remains unclear. miRNAs typically consist of 21–23 nucleotides, are evolutionarily conserved endogenous noncoding RNAs and are vital modulators of gene expression [28]. A recent study has demonstrated that the m7G modification of miRNA inhibits proliferation migration in lung cancer cell lines [31]. Thus, we constructed a prognostic model of LUAD, which combined m7G-related miRNAs signature. A total of 321 m7G-related DEMs were identified in LUAD patients based on the TCGA data. Subsequently, we constructed a prediction model of 28 m7G-related miRNAs signature, contributing to better LUAD survival prediction. In addition, to better understand the immune state and provide a new perspective for clinical treatment, the immune cell infiltration and immune function of the high-risk and low-risk subgroups were further evaluated. Our data, for the first time, revealed the relationship between the m7G-related miRNAs signature and TME, and was helpful in predicting prognosis in patients with LUAD.

Numerous studies have demonstrated the role of miRNAs as biomarkers for carcinogenesis, tumor suppression, diagnosis, and prognosis in LUAD [47]. In this study, a total of 28 miRNAs were included in the risk model, and both the ROC curve and survival curve suggested that the model had a good degree of discrimination, which may provide an important method for the prognostic assessment of LUAD. Among them, 8 m7G-related miRNAs were shown to be associated with NSCLC. A previous study showed that knockdown of NEAT1 promotes apoptosis by sponging miR-153-3p, thereby inhibiting cell proliferation, migration, and invasion in NSCLC [48]. miRNA-32-5p has been reported to inhibit epithelial-mesenchymal transition (EMT) and metastasis in LUAD by targeting SMAD family 3 (*SMAD3*) [49]. In addition, the expression of miR-890 was negatively regulated by small nucleolar host gene 3 (*SNHG3*). Notably, SNHG3 was found to promote the progression of LUAD by targeting miR-890 [50]. It has also been reported that suppression of hsa_circ_0000729 could induce pyroptosis and tumorigenesis in NSCLC cells by targeting miR-1281/FOXO3 [51]. Furthermore, let-7f-1-3p may act as a suppressor gene targeting integrin β1 and enhance doxorubicin’s inhibition on lung cancer cell viability in vitro [52]. Chuang Li et al. showed that miR-665 was significantly up-regulated in NSCLC [53]. Consequently, exosomal miR-665 can regulate the expression of HEY-like protein (*HEYL*), a downstream transcription factor of Notch pathway and promote lung cancer cell invasion and migration [54]. Moreover, up-regulation of miR-579-3p was shown to fuel NSCLC cell proliferation [55]. The other m7G-related miRNAs such as hsa-miR-548t-5p [56], hsa-miR-3922-5p [57], hsa-miR-490-5p [58], hsa-miR-4666a-5p [59], hsa-miR-383-3p [60], hsa-miR-6795-5p [61], hsa-miR-4476 [62], hsa-miR-6825-5p [63], and hsa-miR-4665-3p [64] were shown to play important roles in other tumors. Our findings demonstrated that these m7G-related miRNAs are crucial in the occurrence and development of LUAD. However, the mechanisms of these m7G-related miRNAs in LUAD are still unclear and need further exploration. In addition, their relationship with LUAD prognosis need urgent verification in large clinical samples.

Immunotherapy strategies targeting immune checkpoint proteins, such as programmed death-1 (PD-1) and programmed death ligand-1 (PD-L1), have transformed the treatment paradigm in LUAD over the past decade [65]. Although these results are encouraging, increased immune tolerance is frequently documented in patients with LUAD [66]. Mechanistically, this phenomenon may be associated with the fact that developing LUAD dynamically communicates with the surrounding TME, hijacking and evading host immune surveillance [67]. Therefore, there is a need for more research attention on the interaction between LUAD and immune cells within the TME. According to the GSEA analysis, the PD-1 checkpoint pathway was significantly different between the high-risk and low-risk subgroups. The data suggest that the TME may differ between the high-risk and the low-risk subgroups.

The estimated, immune, and stromal scores between the high-risk and low-risk subgroups were then investigated. Our data showed that all three scores were lower in high-risk patients than in the low-risk group. Due to the insufficient information on immunotherapy in the TCGA-LUAD cohort, the relationship between the estimated score, immune score, and stromal score as well as immunotherapy response could not be well analyzed. Furthermore, we performed ssGSEA analysis to evaluate the correlation between the subgroups and immune infiltration and function. The results showed that activated CD8^+^ T cells, effect or memory CD8^+^ T cells, central memory CD4^+^ T cells, T follicular helper cells, type 1 T helper cells, regulatory T cells, activated B cells, immature B cells, natural killer cells, myeloid-derived suppressor cells, activated dendritic cells, immature dendritic cells, eosinophils, mast cells, monocytes, and neutrophil infiltrated were lower in the high-risk group, while memory B cell infiltrated were higher in the high-risk group. Among them, neoantigen-driven T follicular helper cells and B cells synergistically promoted the responses of anti-tumor CD8^+^ T cells in LUAD [68]. In addition, dendritic cells, known as antigen-presenting cells, have been shown to stimulate the differentiation of T cells to eliminate tumor cells [69], while eosinophils in the metastatic TME promote lymphocyte-mediated antitumor immunity [70]. Activated neutrophils have been reported to interact with T cells in two distinct ways. Several studies reported that peripheral blood neutrophils inhibit antigen-non-specific T cell proliferation by releasing argininase-1 and producing ROS [71,72], while other studies showed that neutrophils can provide antigens and auxiliary signals for T cell activation [73,74]. In addition, Eruslanov et.al indicated that tumor-associated neutrophils can stimulate the proliferation of T cells and IF-γ release in the early stages of lung cancer [75]. A recent study revealed that tumor-associated mast cells (TAMCs) in NSCLC were a group of heterogeneous population with different subsets of CD103 expression, which need further analysis, especially to understand whether the TAMCs are phenotypically and functionally shaped by growing tumors [76]. In addition, myeloid-derived suppressor cells (MDSCs) are important components of the immune suppressive network and could inhibit host protective antitumor immunity [77]. On the other hand, tumor-infiltrating regulatory T cells have been shown to inhibit the response of endogenous cytotoxic T cells in LUAD [78]. These results demonstrate that immune cell infiltration in the TME of LUAD is an extremely complex and dynamic process.

Consistent with this result, the immune function revealed significant differences in the CCR, checkpoint, HLA, T-cell_co-inhibition, T-cell_co-stimulation, and type_II_IFN_response between the high and low-risk subgroups. Defective HLA-I antigen processing and presentation are involved in acquired resistance toward an immune checkpoint inhibitor in lung cancer [79]. We then analyzed the association of immune checkpoint genes between the high and low-risk subgroups. The results showed that the expression of *BTLA, TIGIT, CD28, ICOS, CTLA4, TNFRSF14, CD27, HAVCR2, TNFSF9, CD244, CD48, CD83, CD276* and *CD40LG* was higher in low-risk group patients with LUAD. CTLA-4 is an immunoglobulin superfamily member receptor, mainly expressing on the surface of activated and regulatory T cells, and inhibiting the initiation, activation and migration of T cells [80]. Although the expression of *CTLA4* in NSCLC tumor tissues and cell lines has been reported, its expression in normal bronchial epithelium has not been evaluated [81]. Previous reports showed that anti-CTLA4 antibodies may induce PD-L1 expression in NSCLC with wild-type EGFR and high expression of *CTLA4*, which enhances the efficacy of anti-PD-1 therapy [82]. According to the KEYNOTE-001 clinical trial, high PD-L1 expression was necessary for using pembrolizumab in NSCLC [83]. These findings indicate that patients in the low-risk group may benefit from combined anti-CTLA4 and anti-PD-1 immunotherapy. CheckMate-227, a large phase 3 trial in metastatic or recurrent NSCLC, showed that patients treated with a combination of nivolumab and ipilimumab had significantly longer progression free survival (PFS) compared to those treated with chemotherapy [84]. However, there is no data on specific biomarkers for the combination of anti-PD-1 and anti-CTLA4 therapy in patients with advanced NSCLC. In addition, macrophage-associated immune checkpoints, *CD47* and *LILRB1*, were highly expressed in the low-risk group. CD47 is a ligand of the negative immune checkpoint regulator signal regulatory protein α (SIRPα), which could trigger macrophage-mediated elimination of relapsed NSCLC cells when targeted. Notably, simultaneous targeting of *CD47* and *VEGF* via VEGFR1-SIRPα fusion protein could induce infiltration of macrophages and sensitize NSCLC to antiangiogenic agents and *CD47* blockade [85]. Meanwhile, *LILRB1* is an immunoreceptor tyrosine-based inhibitory motif-containing receptor that binds to MHC class I molecules [86]. In vitro model studies have shown that blocking the LILRB1 signaling pathway can activate macrophage activity against solid tumors [87]. According to these data, we believe that low-risk patients with LUAD may be able to benefit from LILRB1-targeted therapy. Consistent with these results, Amira A. Barkal et al. demonstrated that concurrent intercepting of the MHC class I-LILRB1 signaling axis may fuel macrophages to eliminate tumor cells and indirectly promote the functions of other immune cells [87]. Moreover, it has been reported that miR-665 directly target *CD276* by associating with the CD276 3′-UTR region and mediated the downregulation of *CD276* in breast cancer, which may provide a mechanism for miRNA to participate in the regulation of immune-related genes [88].

We further explored the drug response of patients with LUAD between the high-risk and low-risk subgroups. The data suggested that related signaling pathways such as TKI pathway, HDAC pathway, PI3K/AKT/mTOR pathway and Bcl-2 apoptosis may be involved in regulating m7G-related miRNAs. Clinical trials suggest that afatinib is active in NSCLC tumors harboring specific uncommon EGFR mutations, which include Leu861Gln, Gly719Xaa and Ser768Ile [89]. A randomized phase II study of pemetrexed/cisplatin with or without axitinib showed that although patients with axitinib combinations had non-significant differences in PFS, they exhibited a higher ORR compared to chemotherapy alone in non-squamous NSCLC [90]. A recent phase I trial in NSCLC showed that a combination of avelumab, axitinib, and palbociclib exhibited desribale activity and tolerability in NSCLC [91]. Ibrutinib, an irreversible inhibitor of bruton tyrosine kinase, may be a candidate for the treatment of EGFR-mutated NSCLC, even in erlotinib-resistant tumors [92]. Histone acetylation is one of the post-translational modifications that occurs on DNA-packaging proteins, which often results in increased accessibility of promoter regions and transcription of genes in chromosomal local regions [93]. Many cancers express high levels of HDAC and are more sensitive to HDAC inhibitors [94]. Previous studies have shown that HDAC inhibitors can be used to sensitize EGFR-TKIs in treating NSCLC [95,96]. On the other hand, it has shown that HDAC inhibitors promote chemokines’ expression and enhance T-cell infiltration and response to PD-1-blocking immunotherapy [97]. The data suggest that HDAC can sensitize targeted therapy or immunotherapy in NSCLC, which may provide better solutions for clinical treatment. However, there is a need for multi-center clinical trials to validate this finding.

Although our study highlights several exciting findings, data we extracted were obtained from a public database. Therefore, a real-world prospective cohort study may be needed to validate our risk scoring model. In addition, the nomenclature and annotation of the miRNAs in the included studies were not uniform. For instance, some miRNAs were named according to their origin from the 3′ or 5′ arm, while others refer to the miRNAs based on their relative abundance [98]. In addition, the interactions between these prognostic miRNAs and m7G and the molecular mechanisms in LUAD remain unclear. In-depth analysis of these miRNAs’ biological functions may provide a new perspective to further understand the mechanism of carcinogenesis and therapeutic strategies in LUAD.

## 5. Conclusions

In summary, our study uncovered several key m7G-related miRNAs that could also act as prognostic predictors in LUAD. The novel risk model based on m7G-related miRNAs signature contributed to predicting clinical prognosis and guiding treatment in patients with LUAD. In addition, the study provides new insights into the molecular mechanisms of m7G in LUAD.

## Data Availability

The data from this article can be obtained from the public database The Cancer Genome Atlas (https://portal.gdc.cancer.gov/, accessed on 26 July 2022).

## Supplementary Materials

The following supporting information can be downloaded at: https://www.mdpi.com/article/10.3390/biomedicines11061569/s1. Figure S1: The expression of BUD23 (a), METTL1 (b), RAM (c), RNMT (d), TRMT112 (e) and WDR4 (f) in TCGA-LUAD and normal samples (all *p* < 0.05); Figure S2: The enrichment disease pathways of m7G-related DMEs; Table S1: Clinicopathological features of the LUAD in the TCGA cohort; Table S2: Univariate Cox regression analysis for the m7G-related DMEs and OS of LUAD; Table S3: Oxidative stress-related genes screened out from genecards; Table S4: The drug associated with hsa-miR-890 predicted by RNAactDrug.
