# Supplementary material for: Identification of m7G-Related miRNA Signatures Associated with Prognosis, Oxidative Stress, and Immune Landscape in Lung Adenocarcinoma
Source: Biomedicines. 2023 May 29;11(6):1569. doi: 10.3390/biomedicines11061569 (PMC10295992; doi:10.3390/biomedicines11061569)
Supplement: Supplementary file 1 [file biomedicines-11-01569-s001.zip › Supplementary Tables.pdf]

Supplementary Table

Supplementary Table S1 Clinicopathological features of the LUAD in the TCGA cohort

Clinicopathological features		Number(percent)
Age		151(33.2%)
	≤60	304(66.8%)
	＞60	
Gender		
	Male	204(44.8%)
	Female	251(55.2%)
Stage		
	Stage I	249(54.7%)
	Stage II	108(23.7%)
	Stage III	74(16.3%)
	Stage IV	24(5.3%)
T stage		
	T1	160(35.2%)
	T2	237(52.1%)
	T3	38(8.4%)
	T4	17(3.7%)
	Tx	3(0.6%)
N stage		
	N0	294(64.6%)
	N1	85(18.7%)
	N2	64(14.1%)
	N3	2(0.4%)
	Nx	10(2.2%)
M stage		
	M0	308(67.7%)
	M1	23(5.0%)
	Mx	124(27.3%)
Status		
	Alive	300(65.9%)
	Dead	155(23.1%)
Survival Median(months)		18.8

Supplementary Table S2 Univariate Cox regression analysis for the m7G-related DMEs and OS of LUAD

ID	HR	HR.95L	HR.95H	P value
hsa-miR-548t-5p	1.003774	1.000299	1.00726	0.033257
hsa-let-7f-1-3p	0.136707	0.021108	0.885373	0.036826
hsa-miR-590-3p	1.0028	1.000029	1.005578	0.047675
hsa-miR-718	1.00905	1.002558	1.015584	0.006223

hsa-miR-153-3p	0.999917	0.999843	0.999991	0.028812
hsa-miR-4797-5p	1.146813	1.019358	1.290204	0.022672
hsa-miR-32-5p	1.006073	1.002969	1.009187	0.000123
hsa-miR-3922-5p	1.018626	1.004081	1.033381	0.011902
hsa-miR-490-5p	1.016017	1.004098	1.028078	0.00831
hsa-miR-1281	1.027378	1.008622	1.046483	0.004064
hsa-miR-483-5p	1.001926	1.000404	1.00345	0.013134
hsa-miR-579-3p	1.292089	1.08765	1.534955	0.003545
hsa-miR-645	1.001688	1.000366	1.003011	0.01229
hsa-miR-107	1.000018	1.000001	1.000035	0.033318
hsa-miR-216b-5p	1.006269	1.00087	1.011696	0.022793
hsa-miR-143-5p	1.019173	1.004497	1.034064	0.010279
hsa-miR-4666a-5p	0.881132	0.780349	0.994932	0.041156
hsa-miR-548j-3p	0.367689	0.158104	0.855103	0.020153
hsa-miR-335-5p	1.004613	1.000186	1.00906	0.041114
hsa-miR-4747-5p	1.044223	1.013734	1.075629	0.004207
hsa-miR-3183	1.006004	1.000119	1.011923	0.045542
hsa-miR-383-3p	17.29699	1.112963	268.8193	0.041708
hsa-miR-6854-5p	0.537863	0.296864	0.97451	0.040844
hsa-miR-548u	1.002982	1.000262	1.00571	0.031619
hsa-miR-665	1.072416	1.006339	1.142832	0.031185
hsa-miR-6795-5p	4.79939	1.497727	15.3794	0.008295
hsa-miR-4476	1.2028	1.035942	1.396533	0.015376
hsa-miR-6720-5p	2.575799	1.034336	6.414489	0.042105
hsa-miR-3162-5p	0.757587	0.577716	0.993459	0.044705
hsa-miR-3124-3p	1.018184	1.008203	1.028263	0.000336
hsa-miR-6825-5p	1.265423	1.085848	1.474696	0.002572
hsa-miR-3912-5p	1.19727	1.081911	1.324928	0.000496
hsa-miR-518a-5p	1.005901	1.000976	1.010849	0.018787
hsa-miR-4665-3p	4.127531	1.44402	11.79797	0.008153
hsa-miR-4420	1.388356	1.034049	1.864062	0.029058
hsa-miR-6828-3p	1.732399	1.103934	2.718648	0.016847
hsa-miR-4802-3p	3.138424	1.024784	9.611496	0.045195
hsa-miR-890	1.36975	1.050795	1.78552	0.020002

Supplementary Table S3 Oxidative stress-related genes screened out from genecards

Gene Symbol	Description	Relevance score
NOS3	Nitric Oxide Synthase 3	64.48183
NOS2	Nitric Oxide Synthase 2	58.57753
CPT2	Carnitine Palmitoyltransferase 2	47.16217
NOS1	Nitric Oxide Synthase 1	47.10598

SOD1	Superoxide Dismutase 1	45.70882
CAT	Catalase	45.34208
TNF	Tumor Necrosis Factor	39.16946
NFE2L2	NFE2 Like BZIP Transcription Factor 2	38.07993
HMOX1	Heme Oxygenase 1	37.94664
RYR2	Ryanodine Receptor 2	37.92735
TP53	Tumor Protein P53	37.46856
OXSR1	Oxidative Stress Responsive Kinase 1	35.01617
AIFM1	Apoptosis Inducing Factor Mitochondria Associated 1	34.96143
SOD2	Superoxide Dismutase 2	34.72543
GFM1	G Elongation Factor Mitochondrial 1	33.49869
ELAC2	ElaC Ribonuclease Z 2	32.35224
MAPK14	Mitogen-Activated Protein Kinase 14	32.21059
CARS2	Cysteinyl-TRNA Synthetase 2, Mitochondrial	32.13576
GSR	Glutathione-Disulfide Reductase	32.06275
FARS2	Phenylalanyl-TRNA Synthetase 2, Mitochondrial	32.02698
OSGIN1	Oxidative Stress Induced Growth Inhibitor 1	31.78491
MAPK8	Mitogen-Activated Protein Kinase 8	31.02646
MPO	Myeloperoxidase	30.07912
AARS2	Alanyl-TRNA Synthetase 2, Mitochondrial	29.96805
XDH	Xanthine Dehydrogenase	29.7263
OSER1	Oxidative Stress Responsive Serine Rich 1	29.67412
TXN	Thioredoxin	28.18364
IL6	Interleukin 6	28.14098
MTO1	Mitochondrial TRNA Translation Optimization 1	28.14016
OSGIN2	Oxidative Stress Induced Growth Inhibitor Family Member 2	27.29734
EARS2	Glutamyl-TRNA Synthetase 2, Mitochondrial	27.15174
MAPK1	Mitogen-Activated Protein Kinase 1	26.73636
TUFM	Tu Translation Elongation Factor, Mitochondrial	26.28376
PARK7	Parkinsonism Associated Deglycase	26.04134
VARS2	Valyl-TRNA Synthetase 2, Mitochondrial	25.91225
OLR1	Oxidized Low Density Lipoprotein Receptor 1	25.90836
PNPT1	Polyribonucleotide Nucleotidyltransferase 1	25.53405
G6PD	Glucose-6-Phosphate Dehydrogenase	25.43587
CASQ2	Calsequestrin 2	25.40843
IL1B	Interleukin 1 Beta	25.31738
TSFM	Ts Translation Elongation Factor, Mitochondrial	25.28477
SIRT1	Sirtuin 1	24.82348
MTFMT	Mitochondrial Methionyl-TRNA Formyltransferase	24.77712
CYCS	Cytochrome C, Somatic	24.16202
TXN2	Thioredoxin 2	23.80745

CASP3	Caspase 3	23.75385
NQO1	NAD(P)H Quinone Dehydrogenase 1	23.08655
PON1	Paraoxonase 1	23.08622
GPX1	Glutathione Peroxidase 1	22.64148
PTGS2	Prostaglandin-Endoperoxide Synthase 2	22.4342
APP	Amyloid Beta Precursor Protein	22.36378
CALM1	Calmodulin 1	22.35473
CXCL8	C-X-C Motif Chemokine Ligand 8	22.29916
NOS1AP	Nitric Oxide Synthase 1 Adaptor Protein	22.22334
ALB	Albumin	22.20159
HSPA5	Heat Shock Protein Family A (Hsp70) Member 5	22.11136
CRP	C-Reactive Protein	21.93054
MAP3K5	Mitogen-Activated Protein Kinase Kinase Kinase 5	21.88529
MRPL44	Mitochondrial Ribosomal Protein L44	21.87213
SLC6A4	Solute Carrier Family 6 Member 4	21.81761
CYBA	Cytochrome B-245 Alpha Chain	21.54678
CRH	Corticotropin Releasing Hormone	21.42203
MTRFR	Mitochondrial Translation Release Factor In Rescue	21.39492
ACADVL	Acyl-CoA Dehydrogenase Very Long Chain	21.37822
HADHA	Hydroxyacyl-CoA Dehydrogenase Trifunctional Multienzyme Complex Subunit Alpha	21.33283
POLR1C	RNA Polymerase I And III Subunit C	21.2511
GFM2	GTP Dependent Ribosome Recycling Factor Mitochondrial 2	21.20901
INS	Insulin	21.16934
FOXO3	Forkhead Box O3	21.16576
JUN	Jun Proto-Oncogene, AP-1 Transcription Factor Subunit	21.14929
GSTM1	Glutathione S-Transferase Mu 1	21.11669
GTPBP3	GTP Binding Protein 3, Mitochondrial	21.10775
OGG1	8-Oxoguanine DNA Glycosylase	20.92929
HADHB	Hydroxyacyl-CoA Dehydrogenase Trifunctional Multienzyme Complex Subunit Beta	20.84848
G3BP1	G3BP Stress Granule Assembly Factor 1	20.80472
XBP1	X-Box Binding Protein 1	20.65782
RYR1	Ryanodine Receptor 1	20.56754
DDIT3	DNA Damage Inducible Transcript 3	20.49874
ADPRS	ADP-Ribosylserine Hydrolase	20.36423
GSTP1	Glutathione S-Transferase Pi 1	20.25571
FOXO1	Forkhead Box O1	20.19759
ATF4	Activating Transcription Factor 4	20.16486
PRDX2	Peroxiredoxin 2	20.00371

Supplementary Table S4 The drug associated with hsa-miR-890 predicted by RNAactDrug

miRNA	Drug	Omics	Source	Spearman.stat	Spearman.fdr
hsa-miR-890	Nutlin-3	Expression	CCLE	0.235	0.011
