# Supplementary figures and images for: Identification of m7G-Related miRNA Signatures Associated with Prognosis, Oxidative Stress, and Immune Landscape in Lung Adenocarcinoma
Source: Biomedicines. 2023 May 29;11(6):1569. doi: 10.3390/biomedicines11061569 (PMC10295992; doi:10.3390/biomedicines11061569)

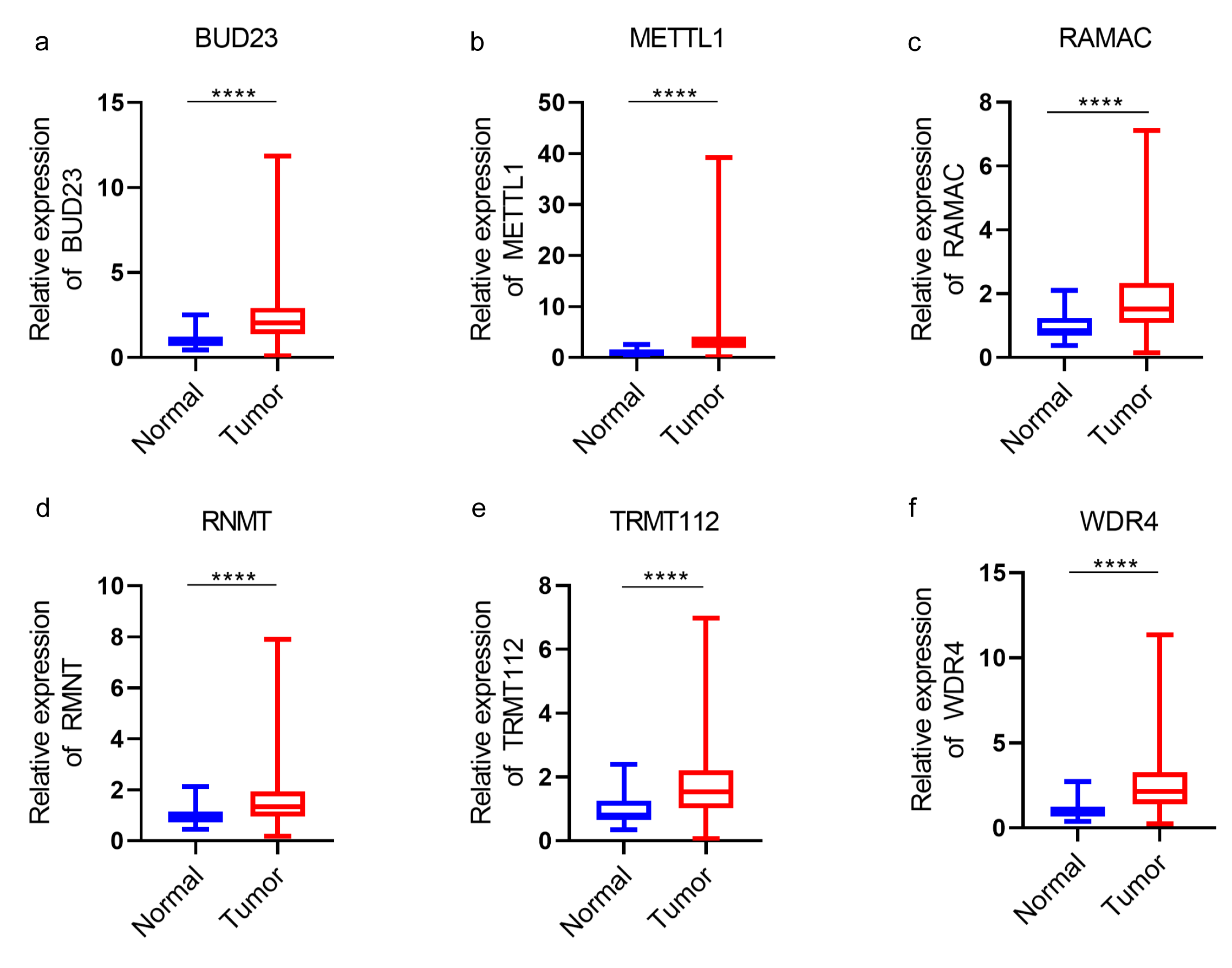

Supplement: Supplementary file 1 [file biomedicines-11-01569-s001.zip › Supplementary Figure S1.tif]

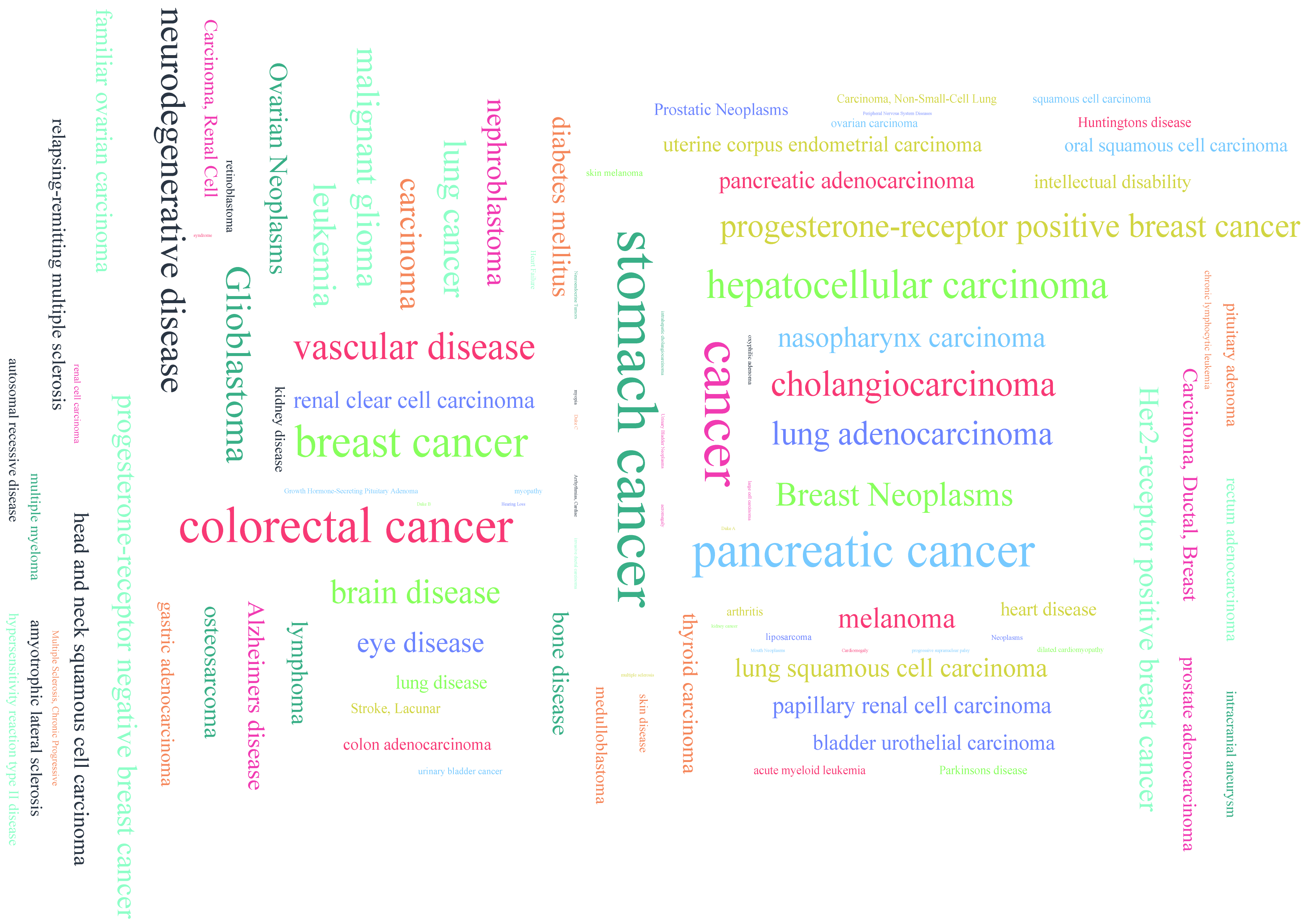

Supplement: Supplementary file 1 [file biomedicines-11-01569-s001.zip › Supplementary Figure S2.tif]
